# Natural disasters and perinatal mental health: what are the impacts on perinatal women and the service system?

**DOI:** 10.1007/s10389-023-01855-y

**Published:** 2023-03-04

**Authors:** Rochelle Helena Hine, Eleanor Mitchell, Lara Whitehead-Annett, Zoe Duncan, Adelle McArdle

**Affiliations:** 1 Monash Rural Health, 15 Sargeant Street, Warragul, Victoria 3820 Australia; 2 Monash Rural Health, Corner Victoria Street & Day Street, Bairnsdale, Victoria 3875 Australia; 3 Monash Rural Health, Northways Rd 3842, Churchill, Victoria 3820 Australia

**Keywords:** Perinatal, Disasters, Mental health, Maternal and child health, Rural

## Abstract

**Aim:**

The perinatal period is characterised by radical change across multiple domains. When it coincides with natural disasters, women and families need targeted support to mitigate the impacts on their birthing and early parenting experiences. Disaster planning in Australia has paid scant attention to the needs of this group. This study aimed to explore rural maternal and child health nurses’ perceptions of how women receiving postnatal care during times of disaster manage mental health and wellbeing issues.

**Subject and methods:**

Eight female maternal and child health nurses (MCHNs) were recruited through purposive sampling across two rural regions of Victoria, Australia. A qualitative design using an online survey followed by in-depth interviews, was underpinned by intersectional feminist theory. Thematic analysis was applied to qualitative data.

**Results:**

Three overarching themes: context of practice, impact of disasters on mothers, and impact of disasters on services were identified. Isolation for mothers was highlighted, necessitating increased provision of emotional support, at a time when service providers themselves were under strain.

**Conclusion:**

Natural disasters exacerbate stressors on perinatal rural women and can impede their access to formal and informal supports, jeopardizing mental health outcomes. Targeted investment in rural perinatal services to enable proactive planning and implementation of disaster strategies is urgently needed to reduce the impact of natural disasters on rural perinatal women and their families.

**Supplementary Information:**

The online version contains supplementary material available at 10.1007/s10389-023-01855-y.

## Introduction

While the impacts of COVID-19 pandemic (hereto referred to as the pandemic) on rural communities have been prominently reported since 2020, the effects of other natural disasters on rural communities have frequently been overshadowed. Drought, flood, and bushfire have had destructive repercussions on the mental health of many rural communities across Australia. Over the summer of 2019–20, devastating bushfires affected over 1.2 million hectares of Victoria, isolating communities and displacing thousands (Victorian Government Department of Environment, Land, Water and Planning 2020). Communities had little time to recover before another disaster — the pandemic — emerged in 2020.

With the pandemic, there has been widespread concern about the mental health of perinatal women (Suwalska et al. 2021). Mental health challenges have occurred as a result of the illness, and also in response to associated events. Lockdowns, social isolation, and loss of income have all had an impact. The loss of access to many community recreation facilities and resources that rural people ordinarily use to maintain wellbeing has further limited coping strategy options (Hine et al. 2020). Statistics indicate family violence (FV) incidents increase during disasters, with well-documented impacts on the mental health of children and adults who are exposed (Campbell 2020; Usher et al. 2020).

The perinatal period is a time of monumental transformation (Maushaut 1997; Mercer 2004). The rural context adds a layer of complexity and potential vulnerability, with challenges around distance and transport, technology (poor internet connectivity), limited service options, and gender stereotypes that influence parenting roles and responsibilities (Shortall 2016). The perinatal period is also highly susceptible to environmental influences (O'Sullivan and Monk 2020), which are further complicated during times of natural disaster. As the needs of perinatal women cannot be postponed until the disaster period has passed, prior consideration and planning is of utmost importance. There is an urgent need to improve understanding of the mental health needs, experiences, and challenges of perinatal women during times of disaster so that systems and services are prepared.

In Australia, maternal and child health nurses (MCHNs) play a pivotal primary care role in supporting the health and wellbeing of women, infants, and families in the early years of the child’s life (Schmied et al. 2014). During the pre-school years, regular MCHN visits are a core component of the support provided to families (Victorian Government Department of Health 2021). As universal service providers, and as an embedded agency within the local government structure, MCHNs are well placed to identify the experiences and needs of women and families at this time and to provide expert guidance for disaster and recovery planning. Opportunities exist for collaborative planning and practice, to ensure the needs of perinatal women and their families are incorporated into disaster and recovery responses.

## Study aims

The research aimed to determine rural MCHN perceptions, experiences, and observations of how rural women receiving postnatal care during times of disaster were managing mental health issues. This study aimed to identify overarching themes pertaining to rurally-based MCHNs in delivering services during disasters, including the 2019/2020 Australian ‘black summer’ bushfires, floods, droughts, and the pandemic.

## Methods

### Study design

This qualitative study was informed by intersectional feminist theory (Rogers and Kelly 2011), underpinned by a critical lens recognising social justice disparities, and the patriarchal social structures influencing women’s experiences (Glenn et al. 2016; Rogers and Kelly 2011). In the context of disasters, these structures include decision-making about responses and deployment of resources (Enarson et al. 2018). There are gender disparities inherent in the way individuals access the physical, social, and environmental resources needed post disaster (Enarson et al. 2018). This study draws on survey and interview data to provide an account of how MCHNs perceive disasters impact the mental health of women in the perinatal period.

Ethics approval was granted by the Monash University Human Research Ethics Committee (application number 26788). Approval for the project was also received from the Victorian Department of Health’s Centre for Evaluation and Research Excellence (CERE) and supported by the Municipal Association of Victoria (MAV), the peak body representing local governments. All participants provided informed consent to participate. Interviews were conducted by a female social worker/academic (RH) with extensive clinical and community mental health experience with another female researcher (ZD, LW).

Practitioners working in the maternal and child health (MCH) sector within three rural regions of Victoria, were invited to participate in a survey and follow-up semi-structured interview. All respondents were MCHNs. The survey collected participant demographic information and explored nurse perceptions and experiences related to the mental health of women and families in the postnatal period who had been accessing their services during the 2019–2021 period. The study was approved by CERE and promoted through the MAV, which were the organisations providing MCH services in the identified areas. Services were contacted directly via telephone and email, and the survey was also advertised through Facebook networks. Recruitment continued until all available contacts were exhausted.

### Data collection

The study was undertaken in two phases; in phase I, an online survey sought information pertaining to the clinicians’ experience and observations within their practice during the period of the disasters. The questions were informed by previous literature in perinatal nursing, disasters, and mental health. Participants were asked to provide demographic and professional experience information (see Table 1).

**Table 1:** Participant characteristics

Pseudonym	Gender	Age	Years of professional experience	Years employed as a MCH nurse	Employment setting	Disasters witnessed
Lauren*	Woman	46	20+	10	Local government	Bushfire, pandemic
Cheryl*	Woman	58	35+	18	Community health	Flood, bushfire, drought, pandemic
Janelle	Woman	41	2+	2	Local government	Pandemic
Tamara	Woman	35	15	4	Local government	Bushfire, drought, pandemic
Kate	Woman	35	15	4	Local government	Bushfire, drought, pandemic
Judy	Woman	43	22	8	Local government	Bushfire, pandemic
Sharon	Woman	55	30	-	ACCHO	Flood, bushfire, pandemic
Sophie	Woman	56	39	3	Community health	Flood, bushfire, drought, pandemic

*Participants who completed a survey and were also interviewed

A quantitative question asked MCHNs to rank mental health and social difficulties identified from the literature and report on observed increases in incidence of severity (see Supplementary Data).

Phase II involved interviews with participants who had completed the survey, via video conferencing. Written informed consent was confirmed prior. Interviews consisted of semi-structured questions and averaged 46 minutes. The questions were developed through coding and thematic analysis of the survey responses. The purpose of the interviews was to gain a deeper understanding of MCHN perceptions of the experiences of women, particularly those from populations who may have experienced additional disadvantages during the disasters.

### Data analysis

Thematic analysis was undertaken by three members of the research team (RH, ZD, LW) to identify key themes from qualitative data collected in both phases. This process followed the six steps articulated by Braun and Clarke (2006): familiarisation with the data, generating initial codes, searching for and reviewing themes, defining and naming themes and report development.

## Results

### Participant demographics

Participants were recruited from three rural regions of Victoria. Eight participants completed the survey. As depicted in Table 1, all participants identified as women and the average age was 46.13 years (range 35–58). Participants were qualified MCHNs, with an average of 22.25 (range 2–39) years of professional experience, including an average of 9.88 (range 2–18) years as MCHNs. All participants had experienced disasters including flood, bushfire, drought, and/or pandemic. Two participants consented to qualitative interviews.

Participants ranked 11 identified mental health and social difficulties from the literature. Two factors — ‘loneliness, isolation or disconnection’ and ‘decreased informal social emotional support’ — were viewed as most critical during disasters (see Table 2 in Supplementary Data).

### Qualitative themes

Themes were grouped under three core headings which were: context of practice, impact of disasters on mothers, and impact of disasters on services. Six main themes emerged from the qualitative analysis of the survey and interview data: (1) context of practice, (2) isolation and struggling, (3) disruption and adaption, (4) the importance of listening, (5) safe and familiar spaces and (6) lack of cohesion.

### Context of practice: rural settings

This theme was a strong undercurrent described in extensive detail within interviews. Participants described working in “small teams” servicing “large geographical areas”, meaning they needed to be flexible and work across programs. Participants expressed strong identities as rural nurses that were unique from the experiences of metropolitan-based nurses, for example, needing to be independent and largely autonomous, “… through COVID I've had to work it all out for myself.”

They also emphasised the significance of alternative identities for both health professional and community members; they were simultaneously farmers, parents, friends and neighbours. This resulted in permeable professional boundaries which at times were challenging and “quite taxing”.

Due to the rural nature of their work and the isolation experienced during disasters, MCHNs identified that mothers had been reaching out to them for increasingly closer personal relationships in ways that didn’t occur prior to the disasters:

*“This other girl that's (sic) very lonely…she's invited us to her baby’s 1-year-old birthday… I wouldn't normally go, I don't normally get invited… I'm the maternal child health nurse, you know, there's got to be a line.”*

MCHNs expressed the enjoyment they derived from confronting unique rural challenges. The variety and multiplicity of the work was a challenge to their practice and learning that they relished. They were proud to identify as autonomous practitioners. MCHNs took pride in their professionalism, honesty, and efficiency in order to manage demands. There were advantages for those who worked as part of a community health service, including access to brokerage funds and opportunities to network with other health professionals:

*… we have really good networks with child protection, so I think that's a real advantage here. Great relationships with the hospital and the mental health services.*

The need to pivot to working from home due to the pandemic necessitated the rapid acquisition of technological skills. Participants remarked on a lack of confidence in using information technology systems. At times, MCHNs’ work included tasks that were typically beyond the normal practice scope such as “facilitat(ing) the NDIS planning, meeting with them... liaising with speech therapy, OT, physio, whatever, mental health services”, in order to meet the needs of the mothers and their families.

MCHNs noted that support for supervision, training, and mentoring was limited, and the ability to take leave was restricted due to the lack of qualified staff in rural areas. If they needed to take a day from clinical practice for professional development purposes or unplanned sick leave, this would “put more stress on other days” so time off was avoided.

## Impact of disasters on mothers

Impacts upon mothers could be divided into two sub-themes, (i) isolation and struggling and (ii) safe and familiar spaces. The theme of ‘isolation and struggling’ describes participants’ observations of the circumstances of many mothers who were under the care of MCHNs during times of disaster. ‘Safe and familiar spaces’ explores the need for appropriate places that mothers could use in times of disasters.

### Isolation and struggling: caring for families facing disadvantage

During the pandemic lockdowns, new mothers had been unable to connect in person with peers and family supports. Compounding this, in rural areas, “reliable internet is not always accessible so zoom catch ups were not that easy”. MCHNs reported that mothers often expressed loneliness, and post bushfires had “lost connection with their friends”. For farmers, partners weren’t a reliable source of emotional support as they were occupied by post-disaster responses.

Women with disabilities, pre-existing mental health challenges, First Nations families, young mothers, women with partners who were incarcerated and those experiencing family violence were identified as needing additional support during times of disaster. MCHNs routinely conducted mental health assessments, in particular for anxiety and depression to enable early identification of women, “at risk due to isolation, lack of access to information, lack of understanding of supports and services, lack of community support.”

MCHNs identified “increased financial aid”, “more place-based services” targeting local needs, “focus on community connections” to reduce loneliness, and enhanced access to mental health services. They also emphasised the importance of face-to-face visits from Family Support and Child Protection Workers, as these had been modified to telephone during the pandemic.

### A safe and familiar space: what is needed during and after disasters

Public evacuation spaces available to communities experiencing disaster were not designed with the specific needs of mothers with infants. Respondents spoke about the possibility of “support hubs that cater to mothers with young babies, including private space for breastfeeding and settling babies, access to shower facilities, access to clean spaces to prepare infant formula.” Although the period of evacuation is usually short, it was described as an intense time that could be traumatic.

*“During a disaster, I'd love to see the (MCH) Centre stay open… And mums, only the mums and babies, can come to… that Centre for an emergency evacuation, rather than up to the basketball stadium… where they can …do their clothes washing, have a shower, use stuff that's in the fridge…”*

Another suggestion was to have a specialised team available to backfill or supplement MCH services, particularly when additional resources were required or when no care was available due to disaster-related events and their ramifications. Mental health support for mothers beyond what MCHNs provide was an identified need. Participants suggested a crisis service immediately after the disaster, with support extending up to 2 years, as the aftermath of a disaster is also potentially gruelling.

## Impact of disasters on services

### Disruption and adaption: MCH service changes during disasters

During disasters, MCH services were often disrupted in affected locations. When staff were personally impacted by disaster, such as bushfires, managing their personal circumstances prevented them from presenting to work. In these situations, “the service literally stopped. So, if there was a newborn baby at the time it was almost impossible to see them and reach them”. During the pandemic lockdowns, MCHNs described being the only health service available within their setting. “The place was empty except for us. And women kept coming…”. This meant an increase in the number and length of appointments, more time spent “counselling” mothers, increased referrals to specialist agencies, and an increased number of women eligible for intensive MCH support through enhanced programs.

MCHNs felt that overall disaster planning was inadequate at their sites, and they lacked support from management to create disaster plans and secure resourcing. Early on in the pandemic, one MCHN proactively sought out what was required for infection control, relating that she had “*personally (gone) to our supermarket and bought hand gel for our centre*,”, concluding*,* “*we've got better stuff to do, haven't we?”*

### The importance of listening: providing emotional care in response to families’ changing needs

Participants highlighted the significance of providing emotional care to address the increased needs of families. They described how “telephone calls were longer” because “people like to tell their story, and they like to tell the whole story”. MCHNs described how mothers sought them out to talk about their experiences, emotions, and concerns; “I'd have mums turning up before the group, for an hour, to chat to me”.

From the perspectives of the participants, mothers felt safe and comfortable opening up to them. Listening and validating became critical skills. Mothers described multiple challenges, such as supporting remote schooling of older children while also “trying to get this baby to settle”.

Mothers who identified as farmers attended MCH-facilitated peer support and education groups as a form of connection but also respite, as it was “the only way they (could legitimately) get away from their farms and their families”. Participants explained that some women had to have a strong rationale to leave the farm as controlling male partners did not tolerate their autonomy. MCHNs recognised this as a form of coercive control, “a sign… of domestic violence and financial abuse”.

Due to pandemic social distancing protocols, it was sometimes more difficult to provide the normally effective and diligent support. MCHNs explained “it's difficult because you can't sit close to parents when they're upset”. Empathetic MCH practice recognises the importance of the practitioner being in close physical proximity to demonstrate compassion when mental health issues are disclosed or when “showing them how to breastfeed”.

### Cohesion and chaos: experiences within the broader service system

This theme recognises the experiences amongst MCHNs within their broader service systems. While support services were offered during times of disaster, the coordination and communication of these support services was sometimes deficient:

*“The area was flooded with support services post bushfire but there was little to no service coordination, and most services were short term. Families struggled to understand which service would best suit which need, and there has been a significant lack of follow-through by many support services.”*

Participants expressed frustration when support services contacted them to scope community need, but often the identified support failed to materialise. The result of multiple requests to provide support only added to workloads, with many organisations requesting separate events to be arranged for them to consult with the community.

An associated issue was the lack of information coordination regarding which local services were operating and what altered arrangements entailed. Participants emphasised resources needed to be disaster-appropriate, relevant and meaningful and they needed to be sustainably and independently resourced and funded rather than adding to the already overwhelming demands of MCH services.

*“…you certainly couldn't use any of those bushfire tools for COVID. They weren't relevant and they weren't really relevant for the drought… it's about having some sort of recognition that these things happen…some tools in the tool bag.”*

Participants strongly advocated for post-disaster coordination to be established, to alleviate the burden on MCHNs for communicating and consulting with multiple government and community-based organisations and service providers. This would enable them to focus on their core responsibilities, and ensuring communities could benefit from additional targeted support.

## Discussion

This study gathered the perspectives of a small sample of rural MCHN on the mental health needs and challenges of perinatal women during disasters. The results provide vivid descriptions of the context of rural MCH practice, including the unique difficulties faced and innovations that services create to overcome them. It is evident rural MCHN juggle multiple roles and that they are an integral part of their rural communities, both in their professional positions and beyond. This study deepens understanding of rural MCHN identities as multifaceted, incorporating their profession, as well as other aspects of their life. MCHNs may also be significantly impacted by disasters personally, with effects on the quality and availability of service delivery.

Analysis identified loneliness, isolation, and disconnection, contributed to by decreased informal social emotional support, as the most significant mental health issues for women in the perinatal period, during disasters. This finding echoes the results of a Canadian study set in an urban environment (Ollivier et al. 2021) where socialisation was a major concern for new mothers, who stated that online peer support groups, while helpful, failed to replace face-to-face contact.

As universal providers, MCH services can offer an adaptive, accessible, and reliable service during and after disasters, and are a valued support within their communities. The structure and framework of the MCH model provides a stable foundation for nurses practicing independently; however, as previous research has identified (Adams et al. 2019; Hooker et al. 2021), there can be a lack of consistency in the implementation of programs, particularly across rural and urban settings. Major challenges are associated with a lack of resources to meet the demands of families and communities and professional isolation — being solo practitioners in remote locations means supervision is not always easy to access. Adams et al. (2019) found that only a third of rurally-based MCHNs received clinical supervision, while 100% of their urban counterparts did. In rural locations, low staffing levels leave communities vulnerable when natural disasters result in an essential universal service often becoming completely unavailable at a time of elevated need. MCHNs found themselves needing to ‘hold’ women and families with inadequate specialised support, through significant mental health challenges, they felt was beyond their scope of practice. Although the available evidence on the nature of rural MCH practice in Australia is scant, what does exist suggests that there are significant differences in the availability of professional development, training, and resources to support nurses to respond to mental health (McCauley et al. 2011), family violence (Hooker et al. 2021) and even child weight issues (Hardy et al. 2019) in rural areas. Geographical health disparities continue to grow, and require dedicated and targeted resourcing to rectify.

This small study reveals some of the social and economic disadvantages impacting on the mental health of rural women experiencing their perinatal period during disasters with the increased demand for food and financial aid noted. Interestingly, the respondents did not rank FV highly as a factor, and this contradicts other research demonstrating FV rates increased during the pandemic (Pfitzner et al. 2022) and during disasters generally (Boxall et al. 2020). FV is also more prevalent in regional, rural, and remote locations (Campo and Tayton 2015) and women may face additional barriers to seeking support and resources as “rural communities are culturally charged social arenas that shape women’s experiences of family violence” (Wendt 2009 p.176). Wendt (2009) found community values such as pride, self-resilience, and privacy, could contribute to supporting cultures of violence against women by discouraging disclosure. It is possible the participants of this study were influenced by these beliefs and are under-estimating the rate and severity of FV. It may also be that the lack of in person contact with women drove the reporting of FV underground. As the literature states, FV increased, but it was not highlighted in this group; maybe it was (dangerously) being missed or the study was too small to reflect changes.

A lack of integration between mental health and MCH services was highlighted as a deficit by participants, and emphasised as an area of practice they would like to see improved. This issue has been highlighted in previous literature (Wakida et al. 2018) including the Royal Commission into Victoria’s Mental Health System (State of Victoria 2021), which recommended community mental health teams provide consultation to practitioners providing care to prospective and new parents.

The results from this study suggest proactive planning for disasters is underdeveloped and currently insufficient to meet the urgent and substantial needs once a disaster strikes. As Malpass et al. (2019) note, “during all phases of a disaster, people evacuating their homes should be in a safe and accessible environment that is equipped to meet their needs” (p. 60). Disaster planning needs to incorporate consideration of the built, social, and cultural environments of places of refuge (Malpass et al. 2019), and services after the disaster has passed. Currently, there is more dedicated planning in Australia for animals during disasters than for infants and children (Gribble et al. 2019). Overall, disaster planning and response coordination must incorporate consideration of the practical and emotional support needs of mothers and infants, especially those with limited resources or conditions that predispose them to additional harms that may compound pre-existing mental health stressors. The implications of failing to provide physical and social environments suitable for perinatal women and families is profound, and could potentially exacerbate the traumatic impact of the disaster. The impact of trauma on both parents and children is long-lasting and has significant costs in terms of quality of life, mental and physical health, and healthcare use (Elfgen et al. 2017; Suardi et al. 2017).

### Research considerations

This study adds to the body of evidence around perinatal mental health during times of disaster, bringing a distinct rural lens. The voices of MCHNs provide deeper understanding of the unique context, challenges, and opportunities of this sector in responding to the needs of perinatal women and their families in times of disaster. The multiple methods of data collection allowed for triangulation of data and incorporation of methods to both quantify the issues, and derive meaning from the accounts of participants.

The small sample of eight is a significant limitation, and suggestive of both the small number of rural MCH nurses as well as barriers to engagement in research amongst rural MCH nurses. The pandemic undoubtedly impacted on recruitment, given the data collection was conducted almost 2 years into the pandemic, and at a time dominated by lockdowns and restrictions.

### Research, policy and practice implications

Further qualitative research exploring experiences of rural mothers in relation to mental health and access to perinatal supports during disasters (beyond COVID) is warranted, to supplement the substantial body of quantitative studies that have emerged during the pandemic. As a group who face additional socioeconomic disadvantages, it is important to specifically consider the needs and experiences of women who have pre-existing mental health challenges, who experience their perinatal phase during a disaster. Investigating the experiences and perspectives of fathers is another need, given that rural cultures of masculinity influence decisions and priorities surrounding both parenting and disaster response.

In Australia, all levels of government policy-makers are engaged in providing services to perinatal women and families. Proactive planning for supporting this vulnerable population in the event of disasters and in the aftermath is urgently required. The timely deployment of targeted resources to supplement existing services is necessary, and consideration must be given to the establishment of safe, supportive evacuation spaces for mothers with infants.

## Conclusion

Natural disasters place relentless strain on already under-resourced publicly funded universal health systems. When many of the usual informal supports and resources for perinatal women are unavailable to them, as occurs during disasters, urgent strategies for building community and connectedness need to be activated. Perinatal women experiencing socioeconomic disadvantage should be prioritised for targeted support.

Pregnancy, birthing, and early parenting cannot be paused during a disaster. The first 1000 days are a time-critical period, with lifelong health and wellbeing implications (Fox et al. 2010). Thus, it is imperative that services, supports and facilities are fully prepared for meeting the needs of perinatal women and their families during disasters.

## Supplementary information

ESM 1 (DOCX 14 kb)

## Data Availability

Data is not publicly available. Requests for data access will be considered on application to Dr Rochelle Hine.
