# Supplementary material for: Natural disasters and perinatal mental health: what are the impacts on perinatal women and the service system?
Source: Z Gesundh Wiss. 2023 Mar 4:1–8. Online ahead of print. doi: 10.1007/s10389-023-01855-y (PMC9984749; doi:10.1007/s10389-023-01855-y)
Supplement: Supplementary file 1 — (DOCX 14 kb) [file 10389_2023_1855_MOESM1_ESM.docx]

**Supplementary Data:** *Ranked mental health or social difficulties.*

**Quantitative data collection**

A quantitative question asked the participants to rank mental health and social difficulties identified from the literature and in order from 1 (most commonly) to 12 (least commonly) observed in or reported by women who had experienced pregnancy and giving birth during disasters. Participants were then asked to rate the observed increase in the incidence or severity of these same mental health or social difficulties during the times of natural disaster.

**Quantitative data analysis**

Quantitative ranked data was analysed using a one-sample Chi Goodness of Fit test^17^ using IBM SPSS Statistics® v. 25 software. The assumption was made that if all factors were equally important they would be equally represented. Each rank was given the score based on their location, with first choice noted as 1 and last choice noted as 11. “Other” was excluded from the analysis as it was often last and left blank. Statistical significance was set at p<0.05.

Chi Goodness of Fit analysis identified two factors - ‘loneliness, isolation or disconnection’ and ‘decreased informal social emotional supports’ as statistically significantly (p<0.001) most highly ranked during disasters.‘Decreased access to psychosocial groups or supports’ and ‘symptoms of anxiety, fear or panic’ also tended to be highly ranked by participants. An additional factor ‘early [hospital] discharge’, was identified by one participant: “[Covid] has increased [the] need for education and support but [it is] not available to the level required as MCH hours are not available to [the] level required to fill [the] gap of a 4 to 5-day hospital stay”.

| **Mental health or social difficulty** | **Average Ranking (overall place)** | **Average perceived increase in incidence during times of natural disaster (overall ranking)** |
| --- | --- | --- |
| Loneliness, isolation or disconnection | 2.75 (Equal 1^st^) | 91.13 (2^nd^) |
| Decreased informal social emotional supports | 2.75 (Equal 1^st^) | 91.63 (3^rd^) |
| Decreased access to psychosocial groups or supports | 5.63 (Equal 2^nd^) | 92.5 (1^st^) |
| Symptoms of anxiety, fear or panic | 5.63 (Equal 2^nd^) | 78.88 (5^th^) |
| Financial stress | 5.88 (3^rd^) | 80.88 (4^th^) |
| Symptoms of depression | 6.13 (4^th^) | 68 (7^th^) |
| Difficulties in attachment and bonding | 6.25 (5^th^) | 61.63 (10^th^) |
| Decreased access to formal support services | 6.63 (6^th^) | 75.38 (6^th^) |
| Difficulties with breastfeeding | 7.63 (Equal 7^th^) | 64.5 (8^th^) |
| Postnatal depression | 7.63 (Equal 7^th^) | 53.88 (11^th^) |
| Family violence | 9.25 (8^th^) | 63.63 (9^th^) |
